# Domain-specific physical activity and depressive symptoms in Korean adults: An isotemporal substitution study using KNHANES data

**DOI:** 10.1371/journal.pone.0338722

**Published:** 2025-12-31

**Authors:** Jungmi Park, Hee-Kyoung Nam, Sung-Il Cho

**Affiliations:** 1 Department of Public Health Sciences, Graduate School of Public Health, Seoul National University, Seoul, Korea; 2 Institute of Aging, Seoul National University, Seoul, Korea; 3 Institute of Health and Environment, Seoul National University, Seoul, Korea; University of Tartu, ESTONIA

## Abstract

**Background:**

Although the mental health benefits of physical activity are well-established, they appear more pronounced when engaging in leisure activities rather than work-based efforts. This phenomenon is often referred to as the physical activity paradox. While leisure activity is often linked to reduced likelihood of depression, the influence of occupational physical activity remains less clearly understood, especially during periods of social disruption such as the COVID-19 pandemic.

**Methods:**

This study analyzed data from 26,454 Korean adults aged 19 years and older who participated in the Korea National Health and Nutrition Examination Survey (KNHANES) in 2014, 2016, 2018, 2020, and 2022. Depressive symptoms were assessed using the Patient Health Questionnaire-9 (PHQ-9), and domain-specific physical activity was measured with the Global Physical Activity Questionnaire (GPAQ). Isotemporal substitution modeling examined associations between reallocating 30 minutes of activity among occupational, transport, and leisure domains and depressive symptoms. Subgroup analyses were conducted across three time periods: pre-pandemic (2014–2018), early pandemic (2020), and later pandemic (2022).

**Results:**

In the full sample, reallocating 30 min from occupational-type moderate-to-vigorous physical activity (MVPA) to leisure MVPA was associated with lower odds of depressive symptoms (odds ratio [OR]: 0.805, 95% confidence interval [CI:] 0.694–0.934), whereas the reverse substitution was linked to higher odds (OR: 1.242, 95% CI: 1.071–1.441). These associations were statistically significant during the pre-pandemic period but did not reach significance during the early or later pandemic phases.

**Conclusions:**

The observed associations between physical activity and depressive symptoms appear to vary by activity type and the social context. The potential mental health benefits of leisure-based activity may be weaker during periods of societal disruption, such as the COVID-19 pandemic. These findings highlight the need for physical activity guidelines that consider both domain-specific patterns and the psychosocial environment in which activity takes place.

## Introduction

Depression represents a major global health concern, impacting approximately 280 million individuals worldwide [1]. South Korea has one of the highest rates of depression among Organisation for Economic Co-operation and Development (OECD) countries, and this has been particularly true following the COVID-19 pandemic [2,3]. Beyond its mental health impact, depression contributes to significant societal costs through increased mortality, reduced productivity, and long-term healthcare expenditures [3,4]. The global economic burden of mental disorders is projected to reach $6 trillion by 2030, underscoring the urgent need for effective prevention strategies [5].

A large body of research has demonstrated that physical activity plays a beneficial role in preventing and managing depression. Consistent evidence shows that regular exercise alleviates depressive symptoms and improves overall mental well-being [6]. However, most of this evidence has focused on leisure-time activity or has not distinguished between activity domains, potentially overlooking important contextual differences in mental health outcomes [7–9]. Recent research highlights that the effects of physical activity may differ across domains, leading to varying implications for mental health [10].

There is consistent evidence supporting the protective effects of leisure-time physical activity, which is associated with reduced depressive symptoms and anxiety [11,12], whereas non-leisure domains show less consistent associations, likely due to differences in the psychosocial and environmental contexts in which the activities occur. Occupational activity, for instance, often involves externally imposed physical demands, low autonomy, repetitive tasks, and limited recovery time, all of which can increase physical strain and psychological stress [13,14]. These features may help explain the non-significant or even positive associations between occupational activity and depression risk that have been reported in previous studies [15]. Transport-related activity also presents mixed findings: while some studies highlight its potential benefits through incidental movement or active commuting, other research, including factors related to commuting conditions and contextual stressors, suggests that these benefits may sometimes be offset [12,16,17]. Although these contextual factors have been discussed as possible explanations for the inconsistent findings across domains, studies directly comparing these domain-specific relationships with depressive symptoms remain scarce.

These mixed findings highlight the need to consider the activity domain when exploring the association between physical activity and mental health. In recognition of this, the 2020 World Health Organization (WHO) Physical Activity Guidelines emphasized the importance of considering activity domains, yet concluded that current evidence remains insufficient to inform domain-specific recommendations [18]. This persistent gap highlights the need for studies directly comparing occupational, transport, and leisure-time activities to better characterize how domain-specific contexts are associated with mental health outcomes.

Given this gap in evidence, it is crucial to move beyond analyses that simply adjust for total physical activity and instead account for time trade-offs among domains. Conventional regression models can identify associations between single domains and depression, but they fail to capture the interdependent nature of daily behaviors, where an increase in one domain necessarily reduces time in another. The isotemporal substitution model (ISM) addresses this limitation by estimating the mental health impact of reallocating time between activity domains while holding total time constant [19]. This framework reflects the finite nature of a 24-hour day and allows for a more realistic assessment of behavioral trade-offs. By modeling these substitutions, ISM helps clarify how shifts in time use between occupational, transport, and leisure activities relate to mental health outcomes, providing evidence to guide domain-specific public health recommendations.

The COVID-19 pandemic fundamentally altered patterns of daily activity, including reductions in commuting, widespread adoption of remote work, closure of gyms and recreational facilities, and increased time spent at home. These disruptions affected not only how much physical activity people engaged in, but also where and how it occurred, turning once-active routines into sedentary ones and limiting opportunities for leisure-based movement [20,21]. Consequently, the mental health benefits traditionally associated with certain types of activity may have diminished or changed. Thus, it is also important to examine this issue across different phases of the pandemic.

Drawing on previous research, we proposed that the relationship between physical activity and depressive symptoms would vary across domains. We expected that leisure-time activity would be associated with lower odds of depressive symptoms, whereas occupational activity would be associated with higher odds. Furthermore, we anticipated that reallocating time from occupational to leisure activity would demonstrate a protective association, while transport activity would show no consistent relationship with depressive symptoms. Given the substantial lifestyle disruptions caused by the COVID-19 pandemic, we expected that these associations would vary across different phases of the pandemic, as daily activity patterns and broader contextual factors influencing mental health had undergone significant changes.

This study aimed to examine how reallocating time among occupational, transport, and leisure activity domains is associated with depressive symptoms in the Korean population, using data from a nationally representative survey (KNHANES) and applying the ISM. We also explored whether these associations differed across three phases of the COVID-19 pandemic: pre-pandemic, early-pandemic, and late-pandemic periods, to reflect changes in daily activity patterns and contextual factors influencing mental health.

## Methods

### Participants and data collection

The data were from the KNHANES, a yearly national health assessment administered by Korea’s Disease Control and Prevention Agency (KDCA) [22]. Given that the Patient Health Questionnaire-9 (PHQ-9) is administered biennially, we utilized information from five survey cycles: 2014, 2016, 2018, 2020, and 2022. From a total of 37,316 participants across these cycles, we selected 26,454 adults aged 19 years and above who had completed the PHQ-9 assessment for our analysis. The full dataset is publicly accessible via the official KNHANES website [23].

### Measurements

Depressive symptoms were assessed using the PHQ-9, a nine-item self-report questionnaire is based on the diagnostic criteria for major depressive episodes as outlined in the Diagnostic and Statistical Manual of Mental Disorders, Fourth Edition (DSM-IV) [24]. Items are rated on a four-point scale ranging from 0 (“not at all”) to 3 (“nearly every day”), yielding total scores between 0 and 27, where higher scores indicate more severe depressive symptoms. Participants were categorized as having depressive symptoms if their PHQ-9 score was ≥ 10. This cutoff has been widely validated as indicating moderate or more severe depressive symptoms, showing approximately 88% sensitivity and specificity when compared with structured clinical interviews for major depressive disorder [25].

Physical activity was measured through the Korean version of the Global Physical Activity Questionnaire (GPAQ), a WHO instrument that has been incorporated into KNHANES since 2014 [26]. The GPAQ assesses physical activity across three domains: occupational, transport, and leisure. In the occupational and leisure domains, activities are categorized by intensity into vigorous (VPA) and moderate physical activity (MPA). Representative examples include running and heavy lifting for VPA, and brisk walking and house cleaning for MPA. For transport-related activities such as walking and cycling, the GPAQ assesses without distinguishing intensity, and according to the WHO GPAQ analysis guide, these activities are considered moderate-intensity activity [27]. The questionnaire also records sedentary behavior (SB) and the frequency and duration of walking.

While device-based measures such as accelerometers are considered a primary tool for objectively assessing physical activity in epidemiological research, they are limited in their ability to capture contextual dimensions. Specifically, accelerometers can objectively quantify the intensity and duration of movement but cannot determine the domain in which the activity occurs, such as during work, transport, or leisure. In contrast, the GPAQ enables domain-specific classification, allowing for more detailed analysis of how physical activity in different contexts may differentially relate to health outcomes such as depressive symptoms [28].

Covariates in the analysis included basic demographic and behavioral factors that have previously been linked to depression. The potential confounding variables examined in this study were survey year, sex, age, marital status, occupational type, education level, household income, smoking status, high-risk drinking, and stress level. Survey year was classified into five categories (2014, 2016, 2018, 2020, and 2022), and sex was categorized as male or female. Age was grouped into five categories: 19–29, 30–39, 40–49, 50–59, and 60 years or older; marital status was classified as married, separated/divorced/widowed, or never married; education level included elementary school or less, middle school, high school, or college or higher; and household income was divided into quartiles (low, lower-middle, upper-middle, and high). Occupational activity was categorized as white collar (managerial/professional and clerical), pink collar (service/sales), blue collar (agricultural/fishery and craft/plant operators), and gray collar (unemployed, students, homemakers). Behavioral factors included smoking status (never smoker, former smoker, or current smoker) and high-risk drinking, defined as consuming seven or more drinks per occasion at least twice per week for men and five or more drinks per occasion at least twice per week for women. Perceived stress level was categorized as high (“very much” or “much”) or low (“a little” or “almost none”).

### Statistical analysis

Descriptive analyses were conducted to examine the characteristics of the study population overall (N = 26,454) and the subgroup with depressive symptoms (n = 1,436), defined by a PHQ-9 score ≥ 10. Categorical variables such as survey year, sex, age, marital status, occupational type, education level, household income, smoking status, high-risk drinking, and stress level were analyzed for group differences using chi-square tests and are presented as frequencies and percentages (n, %). Continuous variables such as SB and physical activity are reported as means with standard errors (SEs). Differences between participants with and without depressive symptoms were evaluated using independent t-tests.

The associations between physical activity domains and depressive symptoms were analyzed using three logistic regression models. First, a single-parameter model was used to examine the independent effect of each domain. Second, a partition model adjusted for all domains simultaneously. Third, an isotemporal substitution model was applied to estimate the effect of reallocating 30 min per day from one activity type to another, holding total activity time constant (S1 Table). A 30-min unit was selected to enhance both the interpretability and practical relevance of the findings [19,29]. Although previous studies have used different substitution intervals, from a few minutes to 2 h per day, the 30-min interval has frequently been adopted as it represents a meaningful and achievable threshold for behavioral modification [30,31]. In the full-sample analysis, all models were adjusted for relevant covariates, including survey year, sex, age, marital status, occupation, education, income, smoking, alcohol use, and stress.

To examine how the COVID-19 pandemic may have influenced the associations between domain-specific physical activity and depressive symptoms, stratified isotemporal substitution analyses were performed across different time periods. Survey years were categorized into three distinct phases: a pre-pandemic period (2014, 2016, 2018), an early COVID-19 phase (2020), and a late COVID-19 phase (2022) [32]. This classification reflects the major shifts in public health measures, social restrictions, and behavioral environments across these time points. Separate isotemporal substitution models were applied for each period using the same set of covariates, except for survey year, which was excluded from the stratified models to avoid overadjustment.

All statistical procedures were carried out using SAS software version 9.4 (SAS Institute Inc., Cary, NC, USA), with p < 0.05 designated as the significance level.

### Ethics statement

This study was exempted from review by Seoul National University’s Institutional Review Board (IRB No. E2409/004–005). It was conducted using data from the KNHANES, a nationally representative survey administered by the KDCA. All KNHANES participants provided written informed consent at the time of the original data collection. The dataset released to researchers is fully anonymized, and the authors had no access to any personally identifiable information. Because this study involved secondary analysis of de-identified public data, the requirement for additional informed consent was waived. The data used in this study were accessed on August 3, 2024.

## Results

Table 1 summarizes the sociodemographic and behavioral characteristics of the study sample (N = 26,454; see table for p values). Among the participants, 1,436 (5.4%) were classified as having depressive symptoms, defined as a PHQ-9 score of 10 or higher. Depressive symptoms were more prevalent among females (6.7%) than males (3.7%). Participants aged 19–29 years showed the highest prevalence of depressive symptoms (7.1%), while those aged 40–49 and 50–59 years had the lowest (4.2%). Marital status was significantly associated with depressive symptoms, with separated, divorced, and widowed individuals showing higher prevalence (10.8%) compared to unmarried (7.3%) and married individuals (3.9%). The prevalence was also highest among gray-collar workers (8.0%) and those with an education level of elementary school or below (8.6%). The prevalence decreased with increasing household income and was higher among current smokers (7.9%) and high-risk drinkers (7.0%). Finally, stress levels showed the largest disparity: 15.7% of participants with high stress were classified as having depressive symptoms, compared to only 1.9% of those with low stress.

**Table 1:** Participant characteristics and demographics.

		Total	With depressive symptoms	P-value
		(N = 26454)	(n = 1436)	
		N	N (%)	
**Year**				
	2014	4844	328 (6.8)	**<0.001**
	2016	5675	346 (6.1)	
	2018	5839	271 (4.6)	
	2020	5323	269 (5.1)	
	2022	4773	222 (4.7)	
**Sex**				
	Male	11500	429 (3.7)	**<0.001**
	Female	14954	1007 (6.7)	
**Age (y)**				
	19 − 29	3337	237 (7.1)	**<0.001**
	30 − 39	4094	235 (5.7)	
	40 − 49	4695	196 (4.2)	
	50 − 59	4955	210 (4.2)	
	≥ 60	9373	558 (6.0)	
**Marital status**				
	Unmarried	4746	345 (7.3)	**<0.001**
	Married	18168	708 (3.9)	
	Separated/Divorced/Widowed	3540	383 (10.8)	
**Occupation type**				
	White	6527	201 (3.1)	**<0.001**
	Pink	3507	184 (5.2)	
	Blue	6033	221 (3.7)	
	Grey	10387	830 (8.0)	
**Education level**				
	≤ Elementary School	5083	438 (8.6)	**<0.001**
	Middle School	2630	165 (6.3)	
	High School	8869	456 (5.1)	
	≥ Undergraduate	9872	377 (3.8)	
**Household Income level**				
	1^st^ (lowest) quartile	4817	523 (10.9)	**<0.001**
	2^nd^ quartile	6449	354 (5.5)	
	3^rd^ quartile	7422	344 (4.6)	
	4^th^ (highest) quartile	7766	215 (2.8)	
**Smoking status**				
	Never	15979	811 (5.1)	**<0.001**
	Former smoker	5807	256 (4.4)	
	Current smoker	4668	369 (7.9)	
**High risk drinking**				
	No	23432	1,225 (5.2)	**<0.001**
	Yes	3022	211 (7.0)	
**Stress level**				
	High	6804	1,070 (15.7)	**<0.001**
	Low	19650	366 (1.9)	

Occupation types were classified into four categories: white-collar (management, professional, and clerical positions), pink collar (service and sales workers), blue collar (agriculture, forestry, fishing, machine operation, craft work, and manual labor positions), and gray collar (unemployed individuals, housewives, and students). All listed variables were adjusted for the analysis. Results meeting the significance criterion of p < 0.05 are presented in bold text.

Table 2 displays the weekly means of SB and physical activity among participants with and without depressive symptoms. Participants with depressive symptoms engaged in significantly more SB (see table for statistics). While MPA and VPA showed no significant between-group differences, total moderate-to-vigorous physical activity (MVPA) was significantly elevated in the group with depressive symptoms relative to those without. Domain-specific analysis revealed that occupational MVPA was higher among participants with depressive symptoms, while transport MPA showed no significant difference between groups. Conversely, leisure MVPA was significantly lower among those with depressive symptoms compared to those without.

**Table 2:** Physical activity status of participants with and without depressive symptoms (2014, 2016, 2018, 2020, 2022).

	Total (N = 26454)	Depressive symptoms		P-value
		With (n = 1436)	Without (n = 25018)	
	Mean ± SE	Mean ± SE	Mean ± SE	
SB	3488.81 ± 14.87	3874.78 ± 55.78	3467.69 ± 15.02	**<0.001**
MPA	212.2 ± 3.06	237.84 ± 15.3	210.80 ± 3.02	0.077
VPA	29.94 ± 1.1	35.11 ± 7.12	29.66 ± 1.08	0.449
MVPA	242.14 ± 3.47	272.95 ± 17.94	240.45 ± 3.43	0.070
Occupational MVPA	56.4 ± 2.52	105.11 ± 14.06	53.73 ± 2.47	**<0.001**
Leisure MVPA	71.64 ± 1.41	50.55 ± 4.86	72.79 ± 1.44	**<0.001**
Transport MPA	114.1 ± 1.71	117.29 ± 6.24	113.93 ± 1.72	0.589

Results meeting the significance criterion of p < 0.05 are presented in bold text.

**Fig 1:**
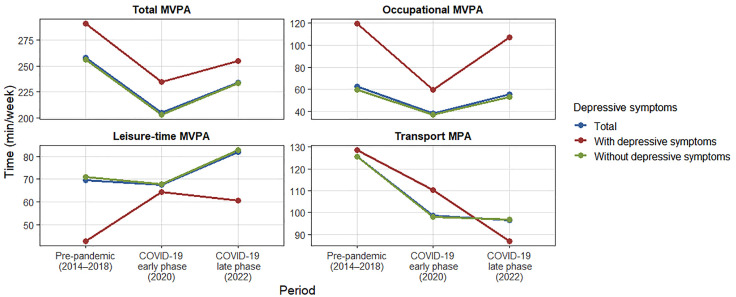
Changes in domain-specific MVPA across pandemic phases by depressive symptoms status.

Fig 1 illustrates the changes in MVPA across the three pandemic phases, disaggregated by depressive symptom status. Total MVPA significantly declined across all groups from pre-pandemic levels to the early pandemic period, with partial recovery observed in the later phase, although activity levels remained below the pre-pandemic baseline. Among participants with depressive symptoms, occupational MVPA showed a marked decline in 2020 followed by a steep increase in 2022, while leisure MVPA remained consistently lower than that of participants without depressive symptoms. Transport MPA declined during the early pandemic and remained relatively stable thereafter in all groups. Notably, participants with depressive symptoms exhibited persistently lower leisure MVPA and higher occupational MVPA compared to those without depressive symptoms throughout the study period. Detailed values are provided in S2–S4 Tables. A same-scale comparison of domain-specific MVPA across pandemic phases is presented in S1 Fig.

### Isotemporal substitution effect of physical activity

Table 3 summarizes the results from an isotemporal substitution analysis that examined the relationships between domain-specific physical activity and depressive symptoms. Three logistic regression approaches were applied: single parameter analysis, partition analysis, and isotemporal substitution analysis. In the single parameter analysis, both SB and occupational MVPA showed significant positive associations with depressive symptoms (SB: OR = 1.025, 95% CI: 1.015–1.035; occupational MVPA: OR = 1.083, 95% CI: 1.043–1.126). Consistent findings were observed in the partition model, where SB (OR = 1.026, 95% CI: 1.016–1.037) and occupational MVPA (OR = 1.100, 95% CI: 1.058–1.144) remained positively associated with depressive symptoms. The isotemporal substitution model revealed several significant findings. Replacing 30 min of SB with leisure MVPA was associated with lower odds of depressive symptoms (OR = 0.863, 95% CI: 0.753–0.989), while substituting SB with occupational MVPA resulted in increased odds (OR = 1.072, 95% CI: 1.031–1.115). Notably, replacing 30 min of occupational MVPA with SB was also associated with reduced odds of depressive symptoms (OR = 0.933, 95% CI: 0.897–0.970). In addition, substituting occupational MVPA with leisure MVPA also showed a protective effect (OR = 0.805, 95% CI: 0.694–0.934), whereas the reverse substitution, replacing leisure MVPA with occupational MVPA, was associated with increased odds of depressive symptoms (OR = 1.242, 95% CI: 1.071–1.441). No significant associations were observed for substitutions involving transport MPA.

**Table 3:** Estimated odds ratios for depressive symptoms by physical activity domain (full sample analysis).

	SB		Occupational MVPA		Leisure MVPA		Transport MPA	
	OR	95% CI	OR	95% CI	OR	95% CI	OR	95% CI
Single-parameter model	**1.025**	**1.015-1.035**	**1.083**	**1.043-1.126**	0.883	0.773-1.009	0.986	0.906-1.073
Partition model	**1.026**	**1.016-1.037**	**1.100**	**1.058-1.144**	0.886	0.773-1.015	1.007	0.926-1.097
Isotemporal substitution model								
Replace SB with	Dropped		**1.072**	**1.031-1.115**	**0.863**	**0.753-0.989**	0.981	0.902-1.068
Replace occupational MVPA with	**0.933**	**0.897-0.970**	Dropped		**0.805**	**0.694-0.934**	0.916	0.837-1.001
Replace leisure MVPA with	**1.159**	**1.011-1.329**	**1.242**	**1.071-1.441**	Dropped		1.137	0.962-1.344
Replace transport MPA with	1.019	0.937-1.108	1.092	0.999-1.195	0.879	0.744-1.039	Dropped	

The single-parameter model adjusted for year, sex, age, marital status, occupation, education, income, smoking, alcohol use, and stress. The partition model used identical covariates while treating each physical activity domain independently without time constraints. The isotemporal substitution model omitted one activity variable from the full model (containing all activities and total activity time) to estimate relative substitution effects. Results meeting the significance criterion of p < 0.05 are presented in bold text.

Tables 4–6 present the results of stratified isotemporal substitution models examining the relationships between domain-specific physical activity and depressive symptoms across three time periods: pre-pandemic (2014, 2016, 2018), early COVID-19 phase (2020), and late COVID-19 phase (2022). Prior to constructing these stratified models, interactions between physical activity domains and time (survey year or pandemic phase) were tested, but none reached statistically significance (all p-values > 0.05). During the pre-pandemic period (Table 4), replacing 30 min per day of occupational MVPA with leisure MVPA was associated with lower odds of depressive symptoms (OR: 0.803, 95% CI: 0.667–0.966). Conversely, replacing leisure MVPA with occupational MVPA was positively associated with depressive symptoms (OR: 1.246, 95% CI: 1.035–1.499). Substituting occupational MVPA with SB was also associated with lower odds of depressive symptoms (OR: 0.926, 95% CI: 0.884–0.969). Other domain substitutions, including those involving transport MPA, were not statistically significant.

**Table 4:** Estimated odds ratios for depressive symptoms by physical activity domain (pre-pandemic: 2014, 2016, 2018).

	SB		Occupational MVPA		Leisure MVPA		Transport MPA	
	OR	95% CI	OR	95% CI	OR	95% CI	OR	95% CI
Single-parameter model	**1.026**	**1.013-1.038**	**1.094**	**1.046-1.145**	0.896	0.760-1.057	0.998	0.901-1.106
Partition model	**1.028**	**1.016-1.041**	**1.111**	**1.061-1.163**	0.892	0.752-1.057	1.017	0.917-1.127
Isotemporal substitution model								
Replace SB with	Dropped		**1.080**	**1.032-1.131**	0.867	0.731-1.029	0.989	0.893-1.095
Replace occupational MVPA with	**0.926**	**0.884-0.969**	Dropped		**0.803**	**0.667-0.966**	0.915	0.822-1.019
Replace leisure MVPA with	1.153	0.971-1.369	**1.246**	**1.035-1.499**	Dropped		1.140	0.923-1.408
Replace transport MPA with	1.011	0.913-1.120	1.092	0.981-1.216	0.877	0.710-1.083	Dropped	

The single-parameter model adjusted for sex, age, marital status, occupation, education, income, smoking, alcohol use, and stress. The partition model used identical covariates while treating each physical activity domain independently without time constraints. The isotemporal substitution model omitted one activity variable from the full model (containing all activities and total time) to estimate relative substitution effects. Results meeting the significance criterion of p < 0.05 are presented in bold text.

In the early COVID-19 phase (Table 5), similar directional patterns were observed, but none of the substitutions reached statistical significance. The substitution of occupational MVPA with leisure MVPA showed a nonsignificant association with lower odds of depressive symptoms as did that with SB. No significant associations were noted for substitutions involving the leisure or transport domains. In the late COVID-19 phase (Table 6), substituting occupational MVPA with leisure MVPA continued to show a negative association with depressive symptoms, although this relationship was not statistically significant (OR: 0.739, 95% CI: 0.489–1.117). Replacing occupational MVPA with SB showed a similar nonsignificant trend (OR: 0.927, 95% CI: 0.842–1.021). The substitution of leisure MVPA with occupational MVPA showed a positive association with depressive symptoms (OR: 1.353, 95% CI: 0.896–2.043), though this also did not meet the threshold for statistical significance. No significant associations were observed for substitutions involving transport MPA in this period. Overall, while the directions of the associations were generally consistent across the three time periods, statistically significant associations were primarily observed in the pre-pandemic period.

**Table 5:** Estimated odds ratios for depressive symptoms by physical activity domain (early pandemic phase; 2020).

	SB		Occupational MVPA		Leisure MVPA		Transport MPA	
	OR	95% CI	OR	95% CI	OR	95% CI	OR	95% CI
Single-parameter model	**1.032**	**1.007-1.057**	1.102	0.985-1.232	1.079	0.829-1.404	1.079	0.893-1.303
Partition model	**1.036**	**1.012-1.061**	**1.125**	**1.002-1.264**	1.083	0.830-1.414	1.107	0.926-1.323
Isotemporal substitution model								
Replace SB with	Dropped		1.086	0.969-1.217	1.046	0.799-1.369	1.069	0.892-1.280
Replace occupational MVPA with	0.921	0.822-1.032	Dropped		0.963	0.705-1.314	0.984	0.810-1.196
Replace leisure MVPA with	0.956	0.730-1.252	1.039	0.761-1.418	Dropped		1.022	0.732-1.427
Replace transport MPA with	0.936	0.782-1.121	1.016	0.836-1.235	0.979	0.701-1.367	Dropped	

The single-parameter model adjusted for sex, age, marital status, occupation, education, income, smoking, alcohol use, and stress. The partition model used identical covariates while treating each physical activity domain independently without time constraints. The isotemporal substitution model omitted one activity variable from the full model (containing all activities and total time) to estimate relative substitution effects. Results meeting the significance criterion of p < 0.05 are presented in bold text.

**Table 6:** Estimated odds ratios for depressive symptoms by physical activity domain (late pandemic phase; 2022).

	SB		Occupational MVPA		Leisure MVPA		Transport MPA	
	OR	95% CI	OR	95% CI	OR	95% CI	OR	95% CI
Single-parameter model	1.008	0.983-1.033	1.071	0.972-1.179	0.808	0.551-1.185	0.934	0.724-1.204
Partition model	1.008	0.984-1.033	1.087	0.989-1.195	0.804	0.548-1.179	0.939	0.728-1.209
Isotemporal substitution model								
Replace SB with	Dropped		1.079	0.979-1.188	0.797	0.544-1.170	0.931	0.720-1.204
Replace occupational MVPA with	0.927	0.842-1.021	Dropped		0.739	0.489-1.117	0.863	0.659-1.132
Replace leisure MVPA with	1.254	0.855-1.840	1.353	0.896-2.043	Dropped		1.168	0.716-1.905
Replace transport MPA with	1.074	0.830-1.389	1.158	0.884-1.518	0.856	0.525-1.397	Dropped	

The single-parameter model adjusted for sex, age, marital status, occupation, education, income, smoking, alcohol use, and stress. The partition model used identical covariates while treating each physical activity domain independently without time constraints. The isotemporal substitution model omitted one activity variable from the full model (containing all activities and total time) to estimate relative substitution effects. Results meeting the significance criterion of p < 0.05 are presented in bold text.

**Fig 2:**
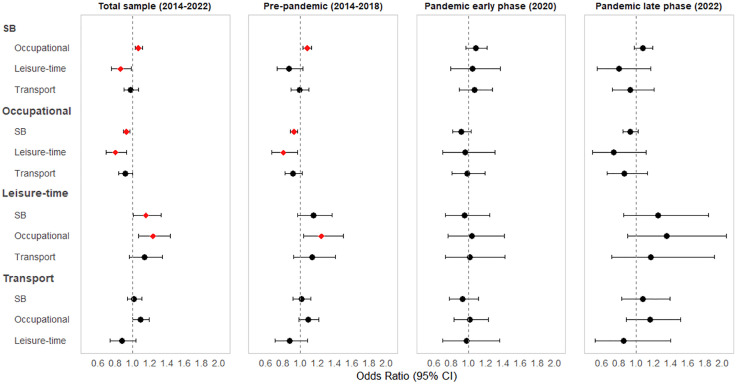
Forest plot of odds ratios for depressive symptoms based on isotemporal substitution of 30 min between physical activity domains: total sample, pre-pandemic, early pandemic phase, and late pandemic phase. *Red diamonds indicate statistically significant associations (p < 0.05). Bolded physical activity types on the y-axis represent the activities that were replaced in the isotemporal substitution models.

Fig 2 illustrates the isotemporal substitution model results using the data from Tables 3–6, presenting forest plots that summarize the associations between domain-specific physical activity substitutions and depressive symptoms across the pandemic periods.

## Discussion

We examined the relationships between SB, domain-specific physical activity, and depressive symptoms using an isotemporal substitution model. Both SB and occupational MVPA were associated with increased odds of depressive symptoms. In particular, substituting 30 min of occupational MVPA with leisure MVPA was associated with decreased odds, while doing the opposite was associated with increased odds. These findings provide empirical support for the physical activity paradox, demonstrating that not all physical activity domains confer equal mental health benefits. While previous studies have largely relied on single-parameter regression models, this study extends the literature by employing an isotemporal substitution approach that captures the behavioral trade-offs between activity domains.

The findings that SB and occupational MVPA were associated with higher odds of depressive symptoms are consistent with previous research. For example, large-scale European studies have reported that occupational-based physical activity and higher levels of SB are both associated with more severe depressive symptoms, particularly among individuals with low levels of leisure-related physical activity [33]. Similarly, evidence from cohort studies that focus on pregnancy have shown that greater SB during pregnancy is associated with increased likelihood of postpartum depression, reinforcing the adverse mental health effects of prolonged SB [34].

Several mechanisms may help explain these associations. Prolonged sedentary behavior can contribute to reduced social interaction, metabolic dysregulation, and inflammatory processes, all of which are linked to depressive symptoms [35–37]. In contrast, occupational MVPA often involves repetitive, externally imposed tasks, limited autonomy, and insufficient recovery, factors that may increase psychological strain and diminish potential mental health benefits [13,38]. In addition to these individual-level pathways, broader contextual factors may also influence how sedentary behavior and occupational MVPA relate to depressive symptoms. Within the labor force, physically demanding or low-control jobs may be more strongly associated with adverse outcomes than white-collar occupations [39]. At a broader level, cross-national differences in labor conditions and workplace protections can influence the extent to which occupational activity becomes a source of strain or resilience [40]. Finally, gender roles may contribute additional complexity, as women often balance occupational demands with caregiving responsibilities [41], potentially amplifying the psychological burden of both sedentary behavior and occupational activity.

Although not statistically significant, leisure MVPA showed a negative association with depressive symptoms, in line with previous research. Population-based studies have demonstrated that higher levels of leisure-based physical activity are associated with lower odds of depressive symptoms, suggesting a potential protective role of such activity against poor mental health outcomes [16]. Similarly, evidence from European adult samples suggests that engagement in leisure-based physical activity is inversely related to the severity of depressive symptoms [33].

Leisure MVPA, typically characterized by voluntary participation, enjoyment, and opportunities for psychological recovery, is associated with improved mental health outcomes [42,43]. In contrast, occupational MVPA often involves repetitive, externally imposed tasks and limited autonomy, which may contribute to increased psychological burden and depressive symptoms [13,44,45].

Additionally, transport MPA did not exhibit consistent or significant associations with depressive symptoms across any of the study periods. This is in line with previous findings showing that the association between active commuting and mental health is often inconclusive. A recent scoping review reported that previous studies have not consistently demonstrated mental health benefits of active commuting [46]. Similarly, previous studies have found that while active commuting is positively associated with physical wellbeing, it is not significantly associated with mental wellbeing [47]. These findings suggest that the mental health effects of transport MPA may be limited or conditional.

One possible reason for this may be that these activities are frequently undertaken out of need rather than personal choice. As such, they may lack essential psychological features such as autonomy, intrinsic motivation, and opportunities for mental restoration, which are widely recognized as key mechanisms in the mental health benefits of leisure-related physical activity [48]. In addition, commuting often involves external stressors such as traffic congestion, time pressure, or adverse weather, which may diminish the potential for psychological benefit. Furthermore, it is possible that the lack of significant associations for transport activity was partly due to measurement limitations. The GPAQ assigns a fixed MET value of 4.0 to all activity for transport, without accounting for variations in actual intensity (e.g., slow vs. brisk walking, casual vs. vigorous cycling) [27]. This simplification may have led to exposure misclassification, potentially attenuating true associations with depressive symptoms.

The isotemporal substitution model clarified how reallocating time between physical activity domains relates to depressive symptoms. Replacing 30 min of occupational MVPA with either leisure MVPA or SB was associated with lower odds of depressive symptoms, while the reverse substitutions showed higher odds. These results reinforce the physical activity paradox and suggest that the mental health impact of physical activity is shaped not just by its domain but also by the activity for which it substitutes [13].

Furthermore, replacing occupational MVPA with SB was associated with a lower odds of depressive symptoms. This suggests that international public health strategies such as the WHO Global Action Plan on Physical Activity and the “Move more, Sit less” message may require contextual adaptation when applied to domains such as occupational MVPA [49]. While SB is often linked to poorer health outcomes, in physically demanding or low-autonomy work environments, engaging in it may serve as a form of psychological recovery when replacing strenuous tasks [13,44,50]. However, further research is needed to clarify which types of SB are most effective for recovery in such occupational contexts.

To explore how the associations between activity domains and depressive symptoms evolved under changing societal conditions, stratified analyses were conducted across three time periods, as outlined above. While the interactions between activity domains and time were not statistically significant, descriptive trends observed in the stratified models suggested potentially meaningful temporal variation. In the pre-pandemic period, replacing occupational MVPA with leisure MVPA or SB was associated with lower odds of depressive symptoms, while the reverse substitutions showed higher odds. These associations were statistically significant and robust, highlighting the distinct mental health implications of physical activity domains under normal social conditions.

During the early phase of the pandemic, patterns seen in previous years were significantly weakened or disrupted. Furthermore, although substituting SB with leisure MVPA was generally associated with lower odds of depressive symptoms, this pattern was less consistent during the pandemic periods. Despite relatively stable levels of leisure MVPA during the early pandemic phase, its mental health benefits may have weakened due to shifts in the way these activities were experienced, including under conditions of reduced enjoyment, limited social interaction, or increased stress [51]. With heightened psychological distress, social isolation, and a blurring of work–life boundaries, leisure-related physical activity may have lost its restorative function, shifting from a voluntary and enjoyable act to a constrained or solitary task [52–54]. This context underscores how the psychological benefits of physical activity are deeply embedded in its social and emotional setting, extending beyond mere physical intensity.

In the late phase of the pandemic (2022), the associations between domain-specific physical activity and depressive symptoms were not statistically significant, despite a general easing of public health restrictions. However, the direction and size of these associations, such as the protective effect of leisure MVPA and the higher odds associated with occupational MVPA, were similar to the patterns observed before the pandemic. Although the paradoxical associations between leisure-related and occupational MVPA weakened during the early pandemic phase, they appeared to restrengthen in the late phase, suggesting that the characteristic domain-specific relationships with depressive symptoms had begun to re-emerge. This trend may reflect a gradual normalization of the social and behavioral contexts that inform the mental health benefits of physical activity. At the same time, ongoing changes in work and daily life, including hybrid work arrangements, economic uncertainty, and disrupted routines, may have continued to interfere with the full restoration of mental health benefits associated with leisure-related physical activity [53–55]. This could help explain why the associations did not reach statistical significance, although the observed trends were consistent with pre-pandemic patterns [56]. These interpretations are based on indirect inference from observational data, and further longitudinal research is needed to determine whether these patterns reflect a lasting change in behavioral context or a temporary adjustment following the pandemic.

The findings underscore the importance of re-evaluating how occupational environments support or inhibit physical activity. Given that occupational MVPA was associated with higher odds of depressive symptoms even before the pandemic, strategies to mitigate its negative impact are warranted. Physical activity in the occupational context often involves physically demanding and nonvoluntary tasks with limited autonomy and insufficient recovery, all of which may contribute to increased psychological strain. Therefore, it is necessary to improve working conditions by increasing autonomy over physical tasks, minimizing repetitive strain, ensuring adequate rest periods, and incorporating opportunities for voluntary and enjoyable physical activity during the workday. Workplaces should also offer supportive environments that include movement or mindfulness breaks and actively encourage leisure-related physical activity to offset the psychological risks associated with occupational activity [57].

This study also offers several innovative contributions. It is among the few to investigate domain-specific physical activity and depressive symptoms using nationally representative data from a non-Western population. By applying an isotemporal substitution model, we were able to estimate the implications of reallocating time between activity domains, moving beyond conventional regression approaches. Furthermore, stratified analyses across pre-pandemic, early pandemic, and late pandemic phases provided novel insights into how societal disruptions may alter the mental health effects of physical activity. Together, these aspects highlight the importance of considering both domain-specific patterns and contextual factors in mental health research.

Our findings further carry practical implications for healthcare professionals and public health practitioners. The results suggest that not all physical activity domains confer equal mental health benefits. While leisure-time activity appears protective, occupational activity may be detrimental under conditions of low autonomy and high physical strain. These findings underscore the importance of workplace adaptations such as increasing autonomy, ensuring adequate recovery time, and promoting voluntary physical activity. For clinicians, encouraging patients to incorporate leisure-based activities such as walking, sports, or recreational exercise may be particularly beneficial for mental health. At the same time, the limited and inconsistent associations of transport-related activity suggest that simply increasing commuting activity may not provide meaningful psychological benefit, emphasizing the need for context-specific counseling and interventions.

Several limitations of this study should be considered. First, the use of a cross-sectional design restricts the capacity to make causal interpretations, as temporal ordering between physical activity and depressive symptoms cannot be established. Second, physical activity and SB were assessed through self-report measures, which may be prone to recall bias and social desirability effects. Third, in the GPAQ, transport-related physical activity was categorized as a single moderate-intensity activity, which may not fully capture variations in actual intensity. This simplification may have introduced some degree of misclassification and could have attenuated the observed associations with depressive symptoms. Fourth, depressive symptoms were assessed using the PHQ-9, which is a validated screening instrument but not a substitute for a clinical diagnosis. Because it relies on self-reported responses, the accuracy of classification may be influenced by social or cultural factors such as mental health stigma or limited awareness and access to care. Therefore, some degree of misclassification cannot be ruled out. Finally, despite adjustment for numerous covariates, unmeasured confounding such as comorbid physical illness or occupational stress may remain.

Future studies should employ longitudinal methodologies and utilize objective measurement devices such as accelerometers, combined with contextual tools such as ecological momentary assessments or time-use diaries. This mixed-method approach can improve the accuracy of domain-specific estimates by balancing measurement objectivity with contextual relevance. Understanding how subjective experiences of activity, including enjoyment, autonomy, and social connection, influence mental health effects may also inform the development of more nuanced, context-sensitive public health guidelines that move beyond activity volume alone.

## Conclusion

This study investigated how the mental health effects of physical activity differ by domain and context. Using an isotemporal substitution model, we found that shifting 30 min from occupational MVPA to leisure MVPA or to SB was associated with decreased odds of depressive symptoms. In contrast, substituting leisure MVPA with occupational MVPA was associated with higher odds. These findings support the physical activity paradox and emphasize that not all activity domains demonstrate equal associations with mental health outcomes. However, stratified analyses showed that these associations were statistically significant in the pre-pandemic period but not during the early or late pandemic periods, suggesting contextual influences on the association between physical activity domains and mental health. Public health guidelines should therefore consider not only the amount but also the domain and psychosocial context of physical activity.

## Supporting information

S1 Table ISM equation. (DOCX)

S2 Table Physical activity status of participants with and without depressive symptoms (2014, 2016, 2018). Abbreviations: SB = sedentary behavior, MPA = moderate physical activity, VPA = vigorous physical activity, MVPA = moderate–vigorous physical activity.
(DOCX)

S3 Table Physical activity status of participants with and without depressive symptoms (2020). Abbreviations: SB = sedentary behavior, MPA = moderate physical activity, VPA = vigorous physical activity, MVPA = moderate–vigorous physical activity.
(DOCX)

S4 Table Physical activity status of participants with and without depressive symptoms (2022). Abbreviations: SB = sedentary behavior, MPA = moderate physical activity, VPA = vigorous physical activity, MVPA = moderate–vigorous physical activity.
(DOCX)

S1 Fig Domain-specific MVPA across pandemic phases by with and without depressive symptoms status (same scale across panels). (DOCX)
