# Supplementary material for: Domain-specific physical activity and depressive symptoms in Korean adults: An isotemporal substitution study using KNHANES data
Source: PLoS One. 2025 Dec 31;20(12):e0338722. doi: 10.1371/journal.pone.0338722 (PMC12818874; doi:10.1371/journal.pone.0338722)
Supplement: S1 Table — (DOCX) [file pone.0338722.s001.docx]

**Supplementary Table 1. ISM equation**

Total behaviors = Sedentary behavior + Occupational MVPA + Leisure MVPA + Transport MPA

$$Depressive symptoms= \beta_{0}+\beta_{1}Occupational MVPA+\beta_{2} Leisure MVPA+\beta_{3} Transport MPA+\beta_{4} Total behaviors$$

(Replacement of sedentary behavior with each remaining factor)

$$Depressive symptoms= \beta_{0}+\beta_{1}Sedentary behavior+ \beta_{2} Leisure MVPA +\beta_{3} Transport MPA+\beta_{4} Total behaviors$$

(Replacement of occupational MVPA with each remaining factor)

$$Depressive symptoms\mathbf{=} \beta_{0}+\beta_{1}Sedentary behavior+ \beta_{2} Occupational MVPA +\beta_{3} Transport MPA+\beta_{4} Total behaviors$$

(Replacement of leisure MVPA with each remaining factor)

$$Depressive symptoms\mathbf{=} \beta_{0}+\beta_{1}Sedentary behavior+ \beta_{2} Occupational MVPA +\beta_{3}\mathrm{Leisure}time MVPA+\beta_{4} Total behaviors$$

(Replacement of transport MPA with each remaining factor)
