# Supplementary material for: Domain-specific physical activity and depressive symptoms in Korean adults: An isotemporal substitution study using KNHANES data
Source: PLoS One. 2025 Dec 31;20(12):e0338722. doi: 10.1371/journal.pone.0338722 (PMC12818874; doi:10.1371/journal.pone.0338722)
Supplement: S2 Table — Abbreviations: SB = sedentary behavior, MPA = moderate physical activity, VPA = vigorous physical activity, MVPA = moderate–vigorous physical activity. (DOCX) [file pone.0338722.s002.docx]

**Supplementary Table 2. Physical activity status of participants with and without depressive symptoms (2014, 2016, 2018)**

|  |  |  | **Total (N = 16,358)** | **Depressive symptoms** | | **P-value** |
| --- | --- | --- | --- | --- | --- | --- |
|  |  |  |  | **With** (n = 945) | **Without** (n = 15,413) |  |
|  |  |  | Mean ± SE | Mean ± SE | Mean ± SE |  |
|  | | | | |  |  |
|  | SB | | 3354.9±19.46 | 3775.10±70.37 | 3330.96±19.49 | <0.001 |
|  | MPA | | 224.8±4.35 | 253.01±21.62 | 223.19±4.32 | 0.169 |
|  | VPA | | 33.18±1.63 | 38.29±10.88 | 32.88±1.60 | 0.623 |
|  | MVPA | | 257.97±4.95 | 291.31±26.05 | 256.07±4.89 | 0.178 |
|  |  | Occupational MVPA | 62.79±3.59 | 119.68±20.04 | 59.55±3.58 | 0.003 |
|  |  | Leisure MVPA | 69.48±1.84 | 42.96±5.56 | 70.99±1.89 | <0.001 |
|  |  | Transport MPA | 125.7±2.43 | 128.67±8.92 | 125.53±2.43 | 0.722 |

SB: sedentary behavior, MPA: moderate physical activity, VPA: vigorous physical activity, MVPA: moderate–vigorous physical activity
