# Supplementary material for: Domain-specific physical activity and depressive symptoms in Korean adults: An isotemporal substitution study using KNHANES data
Source: PLoS One. 2025 Dec 31;20(12):e0338722. doi: 10.1371/journal.pone.0338722 (PMC12818874; doi:10.1371/journal.pone.0338722)
Supplement: S3 Table — Abbreviations: SB = sedentary behavior, MPA = moderate physical activity, VPA = vigorous physical activity, MVPA = moderate–vigorous physical activity. (DOCX) [file pone.0338722.s003.docx]

**Supplementary Table 3. Physical activity status of participants with and without depressive symptoms (2020)**

|  |  |  | **Total (N = 5,323)** | **Depressive symptoms** | | **P-value** |
| --- | --- | --- | --- | --- | --- | --- |
|  |  |  |  | **With** (n = 269) | **Without** (n = 5,054) |  |
|  |  |  | Mean ± SE | Mean ± SE | Mean ± SE |  |
|  | | | | |  |  |
|  | SB | | 3635.14±33.83 | 4180.79±133.65 | 3605.31±34.35 | <0.001 |
|  | MPA | | 182.45±5.46 | 200.85±31.04 | 181.44±5.08 | 0.520 |
|  | VPA | | 22.35±1.66 | 33.64±9.66 | 21.74±1.65 | 0.223 |
|  | MVPA | | 204.80±6.14 | 234.49±32.45 | 203.18±5.78 | 0.321 |
|  |  | Occupational MVPA | 38.64±4.56 | 59.87±25.89 | 37.48±3.92 | 0.354 |
|  |  | Leisure MVPA | 67.56±2.73 | 64.28±11.48 | 67.74±2.80 | 0.769 |
|  |  | Transport MPA | 98.61±3.08 | 110.34±12.64 | 97.97±3.11 | 0.333 |

SB: sedentary behavior, MPA: moderate physical activity, VPA: vigorous physical activity, MVPA: moderate–vigorous physical activity
