# Supplementary material for: Domain-specific physical activity and depressive symptoms in Korean adults: An isotemporal substitution study using KNHANES data
Source: PLoS One. 2025 Dec 31;20(12):e0338722. doi: 10.1371/journal.pone.0338722 (PMC12818874; doi:10.1371/journal.pone.0338722)
Supplement: S4 Table — Abbreviations: SB = sedentary behavior, MPA = moderate physical activity, VPA = vigorous physical activity, MVPA = moderate–vigorous physical activity. (DOCX) [file pone.0338722.s004.docx]

**Supplementary Table 4. Physical activity status of participants with and without depressive symptoms (2022)**

|  |  |  | **Total (N = 4,773)** | **Depressive symptoms** | | **P-value** |
| --- | --- | --- | --- | --- | --- | --- |
|  |  |  |  | **With** (n = 222) | **Without** (n = 4,551) |  |
|  |  |  | Mean ± SE | Mean ± SE | Mean ± SE |  |
|  | | | | |  |  |
|  | SB | | 3725.00±29.76 | 3864.78±120.90 | 3718.25±30.65 | 0.242 |
|  | MPA | | 205.87±5.99 | 228.65±23.74 | 204.77±6.11 | 0.325 |
|  | VPA | | 28.27±1.93 | 26.12±8.66 | 28.37±2.00 | 0.802 |
|  | MVPA | | 234.14±6.82 | 254.77±26.43 | 233.15±6.99 | 0.426 |
|  |  | Occupational MVPA | 55.83±4.92 | 107.13±23.72 | 53.35±4.94 | 0.026 |
|  |  | Leisure MVPA | 81.87±3.40 | 60.53±13.93 | 82.90±3.44 | 0.114 |
|  |  | Transport MPA | 96.45±3.33 | 87.12±8.10 | 96.90±3.43 | 0.249 |

SB: sedentary behavior, MPA: moderate physical activity, VPA: vigorous physical activity, MVPA: moderate–vigorous physical activity
