# Supplementary material for: Domain-specific physical activity and depressive symptoms in Korean adults: An isotemporal substitution study using KNHANES data
Source: PLoS One. 2025 Dec 31;20(12):e0338722. doi: 10.1371/journal.pone.0338722 (PMC12818874; doi:10.1371/journal.pone.0338722)
Supplement: S1 Fig — (DOCX) [file pone.0338722.s005.docx]

**Supplementary Figure 1. Domain-specific MVPA across pandemic phases by with and without depressive symptoms status (same scale across panels)**

**
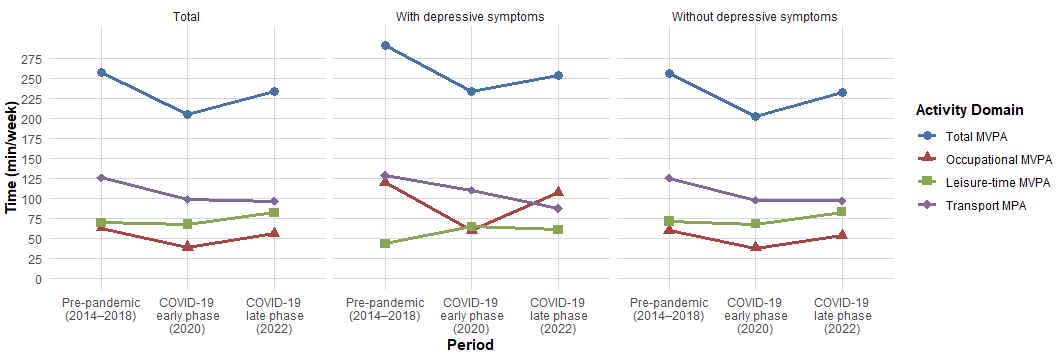
**
